# A Commentary on Chatterjee Et Al. (2018): A Corrected Framework for Group Sparsity in Zero‐Inflated Negative Binomial Models

**DOI:** 10.1002/sim.70356

**Published:** 2025-12-14

**Authors:** Adam Iqbal, Himel Mallick, Emmanuel O. Ogundimu

**Affiliations:** ^1^ Department of Mathematical Sciences Durham University Durham UK; ^2^ Division of Biostatistics, Department of Population Health Sciences Weill Cornell Medicine, Cornell University New York New York USA

**Keywords:** broken adaptive ridge, coordinate‐descent algorithm, group regularization, variable selection, zero‐inflated negative binomial

## Abstract

We reexamine GOOOGLE, the group‐regularized zero‐inflated negative binomial (ZINB) approach of Chatterjee et al. We show that in the released implementation, the tuning parameter is selected using a Bayesian information criterion (BIC) computed on a Gaussian surrogate. Because the unpenalized model fits this surrogate exactly (with zero residual sum of squares), the BIC always favors essentially unpenalized solutions and fails to induce group sparsity. This results in zero group specificity in simulations mirroring the original paper's design. We demonstrate that this issue is resolved by selecting the tuning parameter using the true ZINB log‐likelihood. Furthermore, we propose the fully iterative group broken adaptive ridge (grBAR) estimator as a more robust alternative. Our open‐source R package, group regularization algorithms for zero‐inflated models (GRAZIMs), provides these tools to enable reliable group selection in ZINB models.

## 1 Introduction

Variable selection for zero‐inflated negative binomial (ZINB) models with grouped covariates is important in many biomedical and health applications. The work by Chatterjee et al. [1] made a valuable contribution in *Statistics in Medicine* by proposing GOOOGLE, a framework for applying group‐wise penalties (e.g., group LASSO (grLASSO), group SCAD (grSCAD), group MCP (grMCP)) to ZINB models. The stated goal was to enable simultaneous parameter estimation and selection of covariate groups in both the count and zero‐inflation components.

Methodologically, GOOOGLE relies on a least squares approximation (LSA) to the ZINB log‐likelihood [2], constructed via a Taylor expansion around the unpenalized maximum likelihood estimator (MLE). The resulting weighted least‐squares problem can be solved efficiently using existing group‐descent algorithms.

In this article, we show that while the LSA is a viable computational device, the GOOOGLE model selection step leads to a practical failure of group selection in the settings we study. The published procedure chooses the tuning parameter λ using a Bayesian information criterion (BIC) computed on the *Gaussian surrogate*. As we demonstrate formally, this surrogate is perfectly fit by the unpenalized MLE; consequently, the selection criterion is always minimized at the smallest λ on the path. This yields a dense, nearly unpenalized model and near‐zero group specificity, a behavior confirmed in our replications of the original simulation settings.

This failure is remedied by selecting λ using the *true* ZINB log‐likelihood, which restores the ability of standard group penalties (grLASSO, grSCAD, grMCP) to identify sparse models. Furthermore, we propose the use of the group broken adaptive ridge (grBAR) estimator, a fully iterative scheme that improves robustness by allowing the penalty to influence both the expansion point and curvature of the likelihood approximation. BAR has been applied to GLMs [3, 4] and to survival analysis [5].

Beyond the selection rule, our implementation includes an efficient group coordinate‐descent solver for grLASSO, grSCAD, and grMCP, in which the two intercepts (count and zero) and the dispersion parameter are explicitly updated at every sweep, rather than being held fixed or estimated once via a plug‐in strategy (as in [6], where unpenalized terms are obtained outside the iterative loop and added back after regularization). Updating these unpenalized terms is essential for stable fitting and for avoiding bias in shrinkage propagation in ZINB. For completeness, we also provide group versions of SELO (Seamless $L_{0}$) and SICA (smooth integration of counting and absolute deviation) [7, 8] under the same corrected framework.

## 2 The LSA Framework and the Critical Role of Model Selection

### 2.1 The Least Squares Approximation (LSA) Framework

#### 2.1.1 Model and Notation

Let $Y_{i}$ denote the count for subject i, (i=1,…,n, where n is the number of subjects), with mean $\mu_{i}>0$, structural‐zero probability $p_{i}\in [0,1]$, and dispersion ϕ>0. Under the ZINB model,

$$\begin{array}{cc} & \mathrm{Pr}(Y_{i}=y_{i})=\\ & \;\left\{\begin{array}{ll} p_{i}+(1-p_{i}){\left(\frac{\phi }{\mu_{i}+\phi }\right)}^{\phi },\; & y_{i}=0,\\ (1-p_{i})\frac{\Gamma (\phi +y_{i})}{\Gamma (y_{i}+1)\Gamma (\phi )}{\left(\frac{\mu_{i}}{\mu_{i}+\phi }\right)}^{y_{i}}{\left(\frac{\phi }{\mu_{i}+\phi }\right)}^{\phi },\; & y_{i}\geq 1. \end{array}\right. \end{array}$$

We use log and logit links

$$\log\mu_{i}=x_{i}^{⊤}\beta ,\;\text{logit}(p_{i})=z_{i}^{⊤}\gamma ,$$

with $\beta \in {\mathbb{R}}^{p}$ and $\gamma \in {\mathbb{R}}^{q}$. Let $\theta ={\left(\phi ,\beta^{⊤},\gamma^{⊤}\right)}^{⊤}$ and define the log‐likelihood

$$\begin{array}{cc} \ell (\theta ) & =\overset{n}{\underset{i=1}{\sum }}\left\{1(y_{i}=0)\;\log\left[p_{i}+(1-p_{i}){\left(\frac{\phi }{\mu_{i}+\phi }\right)}^{\phi }\right]+1(y_{i}>0)\right.\\ & \;\times \left[\log(1-p_{i})+\log\left(\frac{\Gamma (\phi +y_{i})}{\Gamma (y_{i}+1)\Gamma (\phi )}\right)+y_{i}\log\left(\frac{\mu_{i}}{\mu_{i}+\phi }\right)\right..\\ & \;\left.\left.+\phi \log\left(\frac{\phi }{\mu_{i}+\phi }\right)\right]\right\}. \end{array}$$

Penalization is applied only to the stacked coefficient vector $\tilde{\theta }={\left(\beta^{⊤},\gamma^{⊤}\right)}^{⊤}$; the two intercepts ($\beta_{0}$ and $\gamma_{0}$) and the dispersion ϕ are unpenalized but updated at every iteration.

Applying group penalties to the ZINB log‐likelihood is computationally demanding. A practical strategy, used by Chatterjee et al. [1], is a one‐step LSA [2]. This involves computing the unpenalized MLE, $\hat{\theta }$ and its associated curvature matrix, ${\hat{\sum }}^{-1}$ (i.e., the observed information), to solve a single penalized quadratic surrogate:

$$\underset{\theta }{\min}\;\frac{1}{2}{\left(\theta -\hat{\theta }\right)}^{⊤}\;{\hat{\sum }}^{-1}\;(\theta -\hat{\theta })+\lambda \overset{K}{\underset{k=1}{\sum }}f(\theta_{A_{k}};\nu ),$$

where K is the total number of groups; $\left\{A_{k}\right\}$ are predefined, nonoverlapping groups of coefficients; for example, in the German healthcare data (Section 4) each cubic polynomial basis for a continuous covariate contributes a three‐coefficient group; f(·) is the group penalty function (e.g., group LASSO); and ν is any additional shape parameter for the penalty. Unpenalized terms like intercepts are handled separately as group zero.

### 2.2 What Goes Wrong With Surrogate‐Based Model Selection

A key algebraic consequence of the one‐step LSA construction is that the *unpenalized* MLE $\hat{\theta }$ is an *exact* least‐squares fit to the surrogate. Let ℓ(θ) denote the ZINB log‐likelihood and $H(\hat{\theta })≻0$ the observed information at $\hat{\theta }$. A second‐order expansion gives

$$-\ell (\theta )\;\approx \;-\ell (\hat{\theta })+\frac{1}{2}{\left(\theta -\hat{\theta }\right)}^{⊤}H(\hat{\theta })(\theta -\hat{\theta }).$$

Writing $H(\hat{\theta })=C^{⊤}C$ with C a symmetric square root and defining $X^{⋆}=C$, $y^{⋆}=C\;\hat{\theta }$, the quadratic term equals $\frac{1}{2}\|y^{⋆}-X^{⋆}\theta {\|}_{2}^{2}$. The unpenalized minimizer is

$${\hat{\theta }}^{\text{OLS}}={\left(X^{⋆⊤}X^{⋆}\right)}^{-1}X^{⋆⊤}y^{⋆}={\left(C^{⊤}C\right)}^{-1}C^{⊤}C\;\hat{\theta }=\hat{\theta },$$

achieving $\text{RSS}(0)=\|y^{⋆}-X^{⋆}\hat{\theta }{\|}_{2}^{2}=0$. Any penalized solution with λ>0 necessarily has RSS(λ)>0.

In the published implementation of GOOOGLE, the tuning parameter is chosen by BIC computed on this Gaussian surrogate (via grpreg [9]). The usual Gaussian‐form BIC is

$$\text{BIC}(\lambda )=n\log\left(\frac{\text{RSS}(\lambda )}{n}\right)+(\log n)\;\text{df}(\lambda )\;\text{(up to a constant),}$$

where $\text{RSS}(\lambda )=\|y^{⋆}-X^{⋆}{\hat{\theta }}_{\lambda }{\|}_{2}^{2}$ and df(λ) is the effective number of free parameters (here taken as the number of active groups, with unpenalized terms such as intercepts and the dispersion parameter counted separately). It starts at −∞ at λ=0 because RSS(0)=0, and then increases strictly for any λ>0. The linear decrease in df(λ) cannot offset the dominating rise in the log term near λ=0. In practical applications of GOOOGLE, RSS(0) is numerically near zero rather than exactly zero, but the conclusion is unchanged: BIC on the surrogate is minimized at the smallest λ, yielding an effectively unpenalized, dense model with near‐zero specificity in practice.

It is important to note that this surrogate BIC is not the BIC‐type selector proposed in the original LSA work [2]. There, the tuning parameter is chosen by minimizing a criterion defined directly on the quadratic LSA loss,

$${\text{BIC}}_{\text{LSA}}(\lambda )=Q({\hat{\theta }}_{\lambda })+\frac{\log n}{n}\;\text{df}(\lambda ),$$

where Q(θ) denotes the LSA quadratic form (e.g., $Q(\theta )={\left(\theta -\hat{\theta }\right)}^{⊤}{\hat{\sum }}^{-1}(\theta -\hat{\theta })$). This criterion operates on a quadratic loss rather than on log{RSS(λ)}, so it does not suffer from any log‐singularity at RSS(0)=0. It has been shown that it yields selection‐consistent, oracle‐efficient estimators under fixed‐p asymptotics [2]. Similar quadratic‐loss BIC rules have since been adopted in other LSA/composite‐quadratic settings, e.g., [10, 11]. Our criticism, therefore, targets the specific surrogate Gaussian BIC used in GOOOGLE, not the LSA framework or these BIC‐type selectors themselves.

### 2.3 The Solution: Select on the True Likelihood and Iterate the Surrogate

We address the issue in two complementary ways.

First, while ${\text{BIC}}_{\text{LSA}}$ is a coherent criterion for the approximate Gaussian surrogate, we adopt the more direct and statistically principled approach of selecting λ on the *true* ZINB model. For standard group penalties (group LASSO/SCAD/MCP), we compute

$$\text{BIC}(\lambda )\;=\;-2\;\ell \left({\hat{\theta }}_{\lambda }\right)+(\log n)\;\text{df}(\lambda ),$$

where ℓ(·) is the ZINB log‐likelihood, ${\hat{\theta }}_{\lambda }$ is re‐estimated for each λ with both sub‐model intercepts and the dispersion parameter updated within the penalized fit. Although ${\hat{\theta }}_{\lambda }$ is obtained using the LSA generated pseudo‐response $y^{⋆}$ and design $X^{⋆}$, the tuning criterion itself is evaluated on the *original* ZINB likelihood. This likelihood‐based BIC is a direct analogue of the standard GLM BIC and has been employed in related two‐equation settings, including zero‐inflated models and sample selection models e.g., [12, 13]. Evaluating each value of λ along the regularization path on the correct likelihood breaks the degeneracy near λ≈0 and restores the ability to remove null groups.

Second, we employ an iterative scheme via the grBAR estimator [14]. At each iteration, a fresh quadratic surrogate of the log‐likelihood is formed around the *current* parameter estimate. This iterative process allows the penalty to influence both the expansion point and curvature of the approximation, enabling robust group deletion at moderate λ values. While grBAR is solved using a numerically stable matrix‐iteration scheme, we note that extending the coordinate‐descent algorithms designed for *individual* BAR [4] to the *group* setting is a nontrivial task due to the nature of block $\ell_{2}$ norms and iteration‐dependent group weights.

A practical implementation difference concerns how unpenalized blocks (the two component intercepts and the dispersion ϕ) are treated during optimization. Because GOOOGLE delegates the surrogate fit to grpreg, intercepts are effectively held fixed within each coordinate‐descent sweep and recovered post hoc by back‐substitution, while ϕ is fixed at its MLE from the full model. In contrast, group regularization algorithms for zero‐inflated models [GRAZIMs] implements the group‐descent solver from scratch and updates these unpenalized blocks at every iteration (treated as group 0 within each majorization‐minimization (MM) pass [15]). Joint updating improves numerical stability and calibration and avoids reliance on post hoc adjustments.

## 3 Empirical Evidence: Simulation Study

To provide empirical evidence, we replicate Simulation II in the original GOOOGLE paper [1], chosen because it better reflects applied analyses with a mix of continuous and categorical covariates. The design has 30 predictors arranged into 11 groups: six continuous variables are expanded via polynomial terms into 10 predictors (forming six groups), and five categorical factors are encoded into 20 dummy variables (forming five groups). Of the 11 groups, three are active in both the count and zero components; the remaining eight are noise. We vary the desired zero‐inflation level (ψ∈0.3,0.4,0.5) and within‐group correlation (ρ∈0,0.4,0.8). We run 1000 Monte Carlo replicates.

For each replicate and setting (n,ψ,ρ) we generate a single i.i.d. sample of size 2n, use the first n observations for training and the remaining n for testing $\left(n_{T}=n_{P}=n;n\in \{200,1000\}\right)$. Models are fitted on the training set. Variable‐selection metrics (sensitivity, specificity, and Matthews correlation coefficient (MCC)) are computed on the training fit and averaged over replicates, with MCC providing a single‐number summary that jointly reflects sensitivity and specificity (1 for perfect recovery, 0 for chance‐level selection). Predictive accuracy (MAE, MASE, and RMSE; [16]) is evaluated on the held‐out test set and summarized by the median over replicates.

Table 1 summarizes group‐selection metrics for all seven penalties at n=200 with moderate correlation (ρ=0.4) for the proposed GRAZIM framework. All penalties recover meaningful sparsity. In the count component, sensitivities lie between 0.57–0.74 (across ψ∈{0.3,0.4,0.5}), while specificities vary by penalty family: grLASSO 0.79–0.82; grSCAD/grMCP 0.89–0.95; and grALASSO/grSELO/grSICA/grBAR ≥0.93. grBAR attains near‐perfect specificity (≥0.995) with the highest MCC (0.64–0.74). The zero component is more challenging: sensitivities fall to 0.12–0.51, with grLASSO and grSCAD particularly affected. grBAR and grALASSO maintain moderate sensitivity (0.47–0.51) while preserving excellent specificity (0.945–1.000), yielding the highest MCCs (0.50–0.62). For predictive accuracy, grALASSO attains the lowest RMSE (303–518), with grBAR close behind (305–528); grSCAD and grMCP are typically 10%–30% higher.

**TABLE 1:** Group variable selection using GRAZIM package for n=200,ρ=0.4.

Penalty function	Count sub‐model			Zero sub‐model			Predictive metrics		
	Sensitivity	Specificity	MCC	Sensitivity	Specificity	MCC	RMSE	MAE	MASE
ψ=0.3									
grLASSO	0.739	0.820	0.553	0.213	0.976	0.263	516.988	228.209	0.936
grALASSO	0.635	0.988	0.709	0.480	0.961	0.549	518.284	224.608	0.917
grSCAD	0.690	0.946	0.689	0.118	0.991	0.152	576.329	280.443	1.153
grMCP	0.734	0.909	0.670	0.215	0.980	0.258	569.729	275.390	1.137
grSELO	0.741	0.942	0.713	0.350	0.990	0.459	552.397	256.341	1.055
grSICA	0.739	0.943	0.714	0.346	0.990	0.456	552.082	256.864	1.056
grBAR	0.660	0.995	0.735	0.478	0.997	0.601	528.141	245.183	0.998
ψ=0.4									
grLASSO	0.720	0.794	0.505	0.268	0.970	0.319	401.592	174.175	1.046
grALASSO	0.621	0.975	0.673	0.495	0.951	0.542	399.176	168.813	1.020
grSCAD	0.675	0.934	0.655	0.161	0.987	0.200	472.467	230.582	1.400
grMCP	0.711	0.897	0.630	0.272	0.976	0.317	460.631	221.002	1.335
grSELO	0.704	0.945	0.682	0.389	0.991	0.491	437.878	198.340	1.194
grSICA	0.703	0.945	0.683	0.388	0.991	0.490	436.282	198.286	1.194
grBAR	0.631	0.995	0.709	0.510	0.998	0.621	399.654	177.975	1.075
ψ=0.5									
grLASSO	0.659	0.791	0.441	0.265	0.966	0.296	307.818	133.130	1.135
grALASSO	0.573	0.958	0.589	0.470	0.945	0.495	303.333	126.506	1.065
grSCAD	0.616	0.929	0.583	0.155	0.986	0.186	393.192	193.909	1.650
grMCP	0.642	0.893	0.554	0.268	0.975	0.294	381.433	177.473	1.530
grSELO	0.636	0.933	0.593	0.381	0.991	0.463	343.397	153.257	1.295
grSICA	0.637	0.933	0.595	0.382	0.991	0.464	343.035	153.217	1.292
grBAR	0.571	0.995	0.640	0.491	0.998	0.586	305.080	129.985	1.107

Table 2 shows the corresponding results at n=200 for the published GOOOGLE implementation. The results indicate near unpenalized fit: (specificity ≤0.16, MCC ≤0.01), which is consistent with the surrogate‐selection issue highlighted in Section 2.2. For prediction, the picture is mixed: at the same ψ, GOOOGLE often achieves lower MAE/MASE but higher RMSE than GRAZIM.

**TABLE 2:** Group variable selection using GOOOGLE implementation when covariates are not assumed to overlap for n=200,ρ=0.4.

Penalty function	Count sub‐model			Zero su‐bmodel			Predictive metrics		
	Sensitivity	Specificity	MCC	Sensitivity	Specificity	MCC	RMSE	MAE	MASE
ψ=0.3									
grLASSO	0.938	0.063	0.001	0.938	0.063	0.001	556.267	179.480	0.743
grSCAD	0.938	0.064	0.003	0.938	0.066	0.007	555.176	179.401	0.743
grMCP	0.938	0.065	0.004	0.938	0.067	0.008	555.176	179.363	0.743
ψ=0.4									
grLASSO	0.923	0.078	0.002	0.923	0.078	0.001	414.701	119.795	0.725
grSCAD	0.923	0.080	0.005	0.923	0.080	0.005	414.355	119.669	0.724
grMCP	0.923	0.082	0.007	0.923	0.081	0.006	414.374	119.669	0.726
ψ=0.5									
grLASSO	0.851	0.151	0.002	0.850	0.150	0.001	307.973	82.068	0.697
grSCAD	0.851	0.155	0.009	0.851	0.152	0.005	309.553	82.130	0.696
grMCP	0.851	0.156	0.011	0.851	0.152	0.004	309.553	82.092	0.699

At n=1000 (Table A1), all GRAZIM methods improve substantially. grBAR, grSELO, and grSICA achieve near‐perfect specificity (0.998–1.000) in both components, and grBAR delivers the top zero‐component MCC (0.776–0.780). GOOOGLE continues to select nearly all groups (Table A2: specificity ≤0.005), indicating that larger n does not remedy the selection‐criterion failure. For prediction, a split pattern persists at n=1000 across ψ, GRAZIM (typically grALASSO, with grBAR close) achieves lower RMSE than GOOOGLE, whereas GOOOGLE often attains slightly lower MAE/MASE.

**FIGURE B1:**
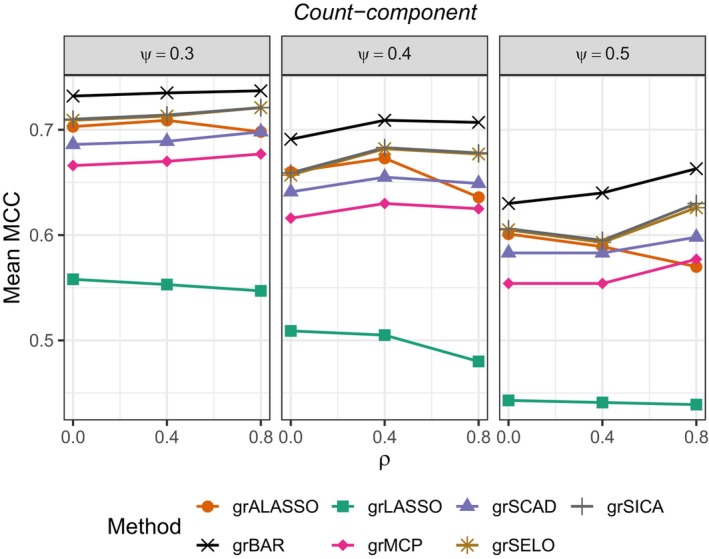
Effect of covariate correlation (ρ) on group MCC by ψ when n=200.

**FIGURE B2:**
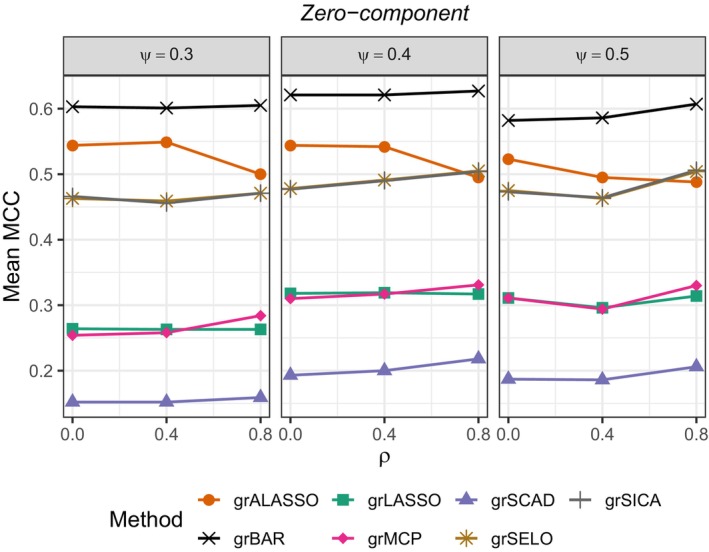
Effect of covariate correlation (ρ) on group MCC by ψ when n=200.

We also investigate the impact of the combined effect of correlation among covariates ρ and ψ on the proposed methods in GRAZIM. Figures B1 and B2 show that grBAR maintains the highest and most stable MCC across ρ∈{0,0.4,0.8} with only mild degradation as ψ increases.

**FIGURE B3:**
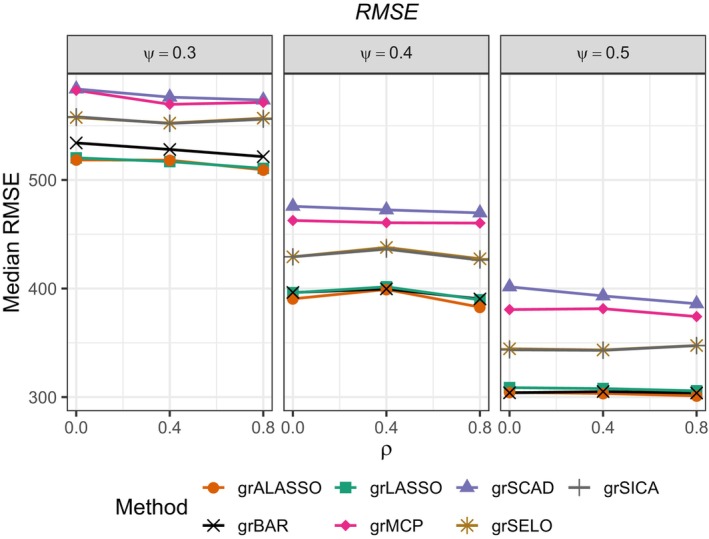
Effect of covariate correlation (ρ) on RMSE by ψ when n=200.

**FIGURE B4:**
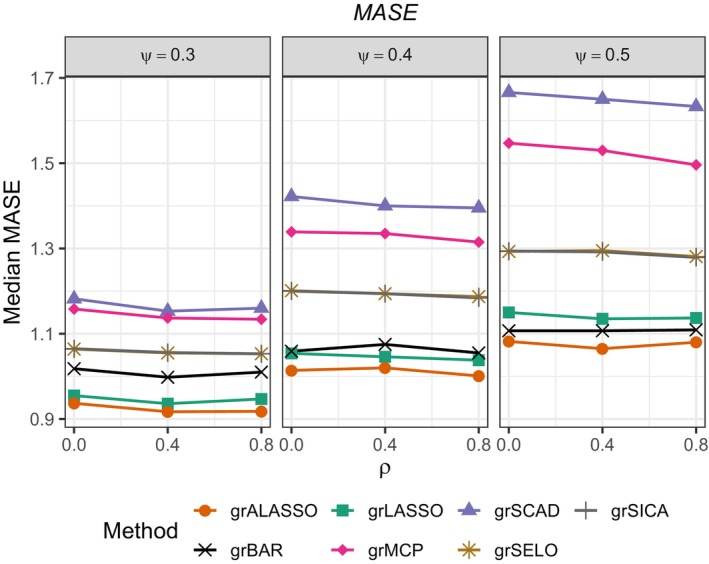
Effect of covariate correlation (ρ) on MASE by ψ when n=200.

Predictive results (Figures B3 and B4) show that grALASSO consistently achieves the lowest RMSE and MASE, with grBAR and grLASSO within ∼2%, while grSCAD/grMCP lag by 10%–15%.

## 4 Reanalysis of the German Healthcare Demand Data

**FIGURE C1:**
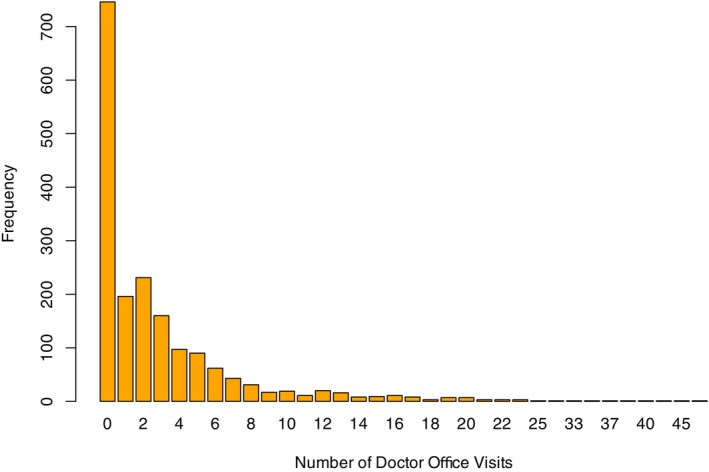
Number of doctor office visits in the German healthcare demand data.

We further illustrate the practical implications by reanalyzing the German healthcare demand dataset used in the original paper. The goal is to model the number of doctor office visits (see Figure C1 for its empirical distribution) using 14 groups of predictors (five polynomial triplets and nine singletons). Chatterjee et al. [1] reported coefficient estimates in their Tables 4 and 5 that show their group LASSO, SCAD, and MCP methods selected nearly all 14 predictor groups. Our reanalysis confirms this behavior for the GOOOGLE implementation (see Table 3), yielding a saturated, hard‐to‐interpret model.

**TABLE 3:** German healthcare demand (doctor visits): number of selected groups (out of 14) in each sub‐model.

Method	Count (out of 14)	Zero (out of 14)
GOOOGLE (grLASSO)^a^	14	14
GOOOGLE (grSCAD)	14	14
GOOOGLE (grMCP)	14	14
GRAZIM (grLASSO)^b^	9	4
GRAZIM (grALASSO)	4	5
GRAZIM (grSCAD)	6	3
GRAZIM (grMCP)	5	2
GRAZIM (grSELO)	4	2
GRAZIM (grSICA)	4	2
GRAZIM (grBAR)	4	2

*Note:* “Groups” refers to the 14 unique predictors; polynomial triplets (e.g., *age1–age3*) count as one group (see Table C1).

^a^ λ chosen by BIC on the Gaussian surrogate (released GOOOGLE code).

^b^ λ chosen by BIC on the true ZINB log‐likelihood. Intercepts and dispersion are unpenalized and updated each iteration.

Under the corrected selection rule based on the true ZINB log‐likelihood (see Table 3), GRAZIM (grLASSO) already reduces model size substantially (9 of 14 groups in the count part and 4 of 14 in the zero part) and improves out‐of‐sample prediction error by 19.5% compared to GOOOGLE (grLASSO). The iterative GRAZIM (grBAR) achieves even greater sparsity (4 of 14 in the count part and 2 of 14 in the zero part) while attaining the lowest prediction error across all methods.

**FIGURE C2:**
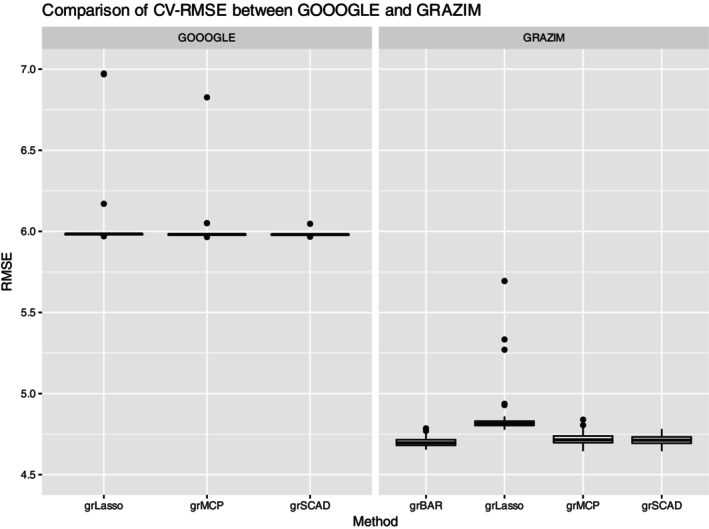
Comparison of CV‐RMSE between GOOOGLE and GRAZIM for 100 replicates of fivefold cross‐validation splits. For grLASSO, grSCAD and grMCP, the GOOOGLE implementation is on the left and the GRAZIM implementation on the right. We cut off several instances where GOOOGLE had RMSE >7 for clarity purposes.

As Table 4 demonstrates, we used fivefold cross‐validation repeated 100 times with identical folds across methods; for each split we predicted $ŷ=𝔼(Y|X)=(1-\hat{p})\hat{\mu }$ and summarized test error with RMSE, MAE, and MASE (medians over repeats). GRAZIM (grBAR) reduces RMSE by 21.5% relative to GOOOGLE (grLASSO) (4.696 vs. 5.984) and achieves competitive MAE (2.677) and MASE (0.863) values. To further visualize the reduction in predictive error, Figure C2 (Appendix C) presents boxplots of the fivefold CV‐RMSE across all 100 replicates. These plots show that, for grLASSO, grSCAD, and grMCP, the entire distribution of RMSE under GRAZIM is markedly lower and more stable than for the corresponding GOOOGLE implementations (with several extreme GOOOGLE RMSE values exceeding 7, truncated in the figure). The grBAR model highlights a concise, scientifically plausible set of drivers (**count**): age, health satisfaction, self‐employed, civil servant; (**zero**): health satisfaction, number of children. These same variables are selected by grSELO and grSICA (both achieving RMSE of approximately 4.71), demonstrating consistency across different penalty functions. Notably, even the benchmark EM LASSO method (RMSE = 4.743) substantially outperforms all GOOOGLE variants, confirming that the failure to perform variable selection on the true likelihood in the original implementation harms both interpretability and predictive performance.

**TABLE 4:** MAE, MASE, and RMSE for the German healthcare demand data (fivefold CV, median over repeats).

Penalty/method	MAE	MASE	RMSE
GRAZIM (corrected selection on true ZINB likelihood)			
grLASSO	2.633	0.848	4.816
grALASSO	2.668	0.860	4.852
grSCAD	2.662	0.858	4.713
grMCP	2.667	0.859	4.715
grSELO	2.645	0.852	4.707
grSICA	2.642	0.851	4.711
grBAR	2.677	0.863	4.696
GOOOGLE (surrogate‐based selection)			
grLASSO	2.949	0.950	5.984
grSCAD	2.947	0.950	5.981
grMCP	2.947	0.950	5.982
EM LASSO^a^			
EM LASSO	2.650	0.854	4.743

^a^ EM LASSO = Expectation–Maximization Least Absolute Shrinkage and Selection Operator; see [17].

## 5 Conclusion

Group selection for ZINB models is a critical task for applied researchers. In this commentary, we diagnosed a subtle but critical flaw in the published GOOOGLE implementation: its reliance on a BIC computed on a Gaussian surrogate effectively prevents it from inducing sparsity in finite samples. We emphasize that our critique concerns the surrogate‐based BIC used in the released GOOOGLE implementation, not the underlying LSA methodology or the theoretical properties derived in Chatterjee et al. [1]. We demonstrated that this is corrected by selecting the tuning parameter based on the true ZINB log‐likelihood, which restores selection capabilities. Furthermore, we proposed the fully iterative grBAR estimator as a novel and highly robust alternative.

Our empirical evidence from a challenging simulation with 30 mixed covariates highlighted the superiority of this corrected approach. The grBAR estimator proved particularly effective, delivering the most balanced and stable selection accuracy (i.e., highest MCC) even at small sample sizes (n=200). We also noted a key statistical pattern: the flawed GOOOGLE models, by retaining all variables, achieved a lower MAE/MASE on the single test split, while our sparse GRAZIM fits had a superior (lower) RMSE. This suggests GOOOGLE's dense models are prone to large, outlier errors, which RMSE penalizes heavily. As noted by [18], this is a classic bias‐variance trade‐off on a single split. Crucially, this artifact disappeared in our real‐data reanalysis (Section 4, Table 4), where robust 100 repeats of fivefold cross‐validation confirmed the GRAZIM framework is unambiguously superior, outperforming GOOOGLE on all metrics: RMSE (21.5% reduction), MAE (9.2% reduction), and MASE (9.2% reduction). The GRAZIM R package is available at: https://github.com/adam‐iqbal/grazim.

A further practical advantage of GRAZIM is that it jointly updates unpenalized terms during optimization, improving numerical stability and avoiding post hoc intercept recovery. The framework provides additional flexibility through implementations for Zero‐Inflated Poisson (ZIP) models [19] and model selection via either AIC or BIC, making it a comprehensive and reliable tool for practitioners working with zero‐inflated count data.

Several directions for methodological extension warrant further investigation. Overlapping‐group and hierarchical structures could be incorporated by combining adaptive‐ridge weights with fusion penalties or graph‐based total variation, thereby facilitating applications such as pathway‐informed genomics and spatial random‐effects modeling. The majorization and reweighting framework underlying grBAR is also readily adaptable to other semicontinuous models, including hurdle models and ZIP regression, with the potential for similarly strong performance in terms of support recovery and parsimony.

## Funding

This work was supported by the Wellcome Trust (Grant No. 314221/Z/24/Z) and the Engineering and Physical Sciences Research Council (Grant No. EP/V521917/1).

## Conflicts of Interest

The authors declare no conflicts of interest.

## Supporting information


**Data S1.** Supporting Information.

## Data Availability

The data that support the findings of this study are available on request from the corresponding author. The data are not publicly available due to privacy or ethical restrictions.

## Appendix A Additional Simulation Results

**TABLE A1:** Group variable selection using GRAZIM package for n=1000,ρ=0.4.

Penalty function	Count sub‐model			Zero sub‐model			Predictive metrics		
	Sensitivity	Specificity	MCC	Sensitivity	Specificity	MCC	RMSE	MAE	MASE
ψ=0.3									
grLASSO	0.982	0.846	0.785	0.751	0.910	0.688	570.865	238.436	0.945
grALASSO	0.741	0.999	0.820	0.686	0.985	0.758	567.728	235.282	0.929
grSCAD	0.988	0.947	0.915	0.746	0.946	0.738	571.364	240.462	0.950
grMCP	0.965	0.977	0.941	0.708	0.976	0.757	572.976	238.414	0.942
grSELO	0.927	0.998	0.946	0.682	0.997	0.776	576.259	239.293	0.945
grSICA	0.927	0.998	0.946	0.683	0.997	0.776	576.264	239.297	0.945
grBAR	0.907	0.999	0.934	0.677	0.999	0.776	572.496	237.925	0.941
ψ=0.4									
grLASSO	0.961	0.850	0.772	0.749	0.912	0.689	436.463	175.711	1.012
grALASSO	0.727	0.999	0.810	0.680	0.990	0.761	435.004	172.427	0.993
grSCAD	0.966	0.959	0.916	0.725	0.960	0.743	436.860	176.378	1.013
grMCP	0.912	0.984	0.915	0.695	0.986	0.765	437.500	174.821	1.004
grSELO	0.877	0.998	0.912	0.680	0.998	0.775	438.794	175.126	1.002
grSICA	0.877	0.998	0.912	0.680	0.998	0.775	438.803	175.111	1.002
grBAR	0.846	0.999	0.892	0.676	0.999	0.775	438.020	173.775	0.997
ψ=0.5									
grLASSO	0.932	0.844	0.742	0.744	0.914	0.687	348.609	131.829	1.047
grALASSO	0.705	0.999	0.795	0.676	0.994	0.765	347.594	129.117	1.021
grSCAD	0.927	0.965	0.897	0.712	0.970	0.749	348.576	132.225	1.045
grMCP	0.845	0.986	0.872	0.686	0.989	0.763	349.025	129.982	1.033
grSELO	0.827	0.998	0.877	0.678	0.998	0.775	350.834	129.849	1.034
grSICA	0.827	0.998	0.877	0.678	0.998	0.775	350.846	129.839	1.034
grBAR	0.795	0.999	0.857	0.677	1.000	0.776	345.671	128.923	1.023

**TABLE A2:** Group variable selection using GOOOGLE implementation when covariates are not assumed to overlap for n=1000,ρ=0.4.

Penalty function	Count sub‐model			Zero sub‐model			Predictive metrics		
	Sensitivity	Specificity	MCC	Sensitivity	Specificity	MCC	RMSE	MAE	MASE
ψ=0.3									
grLASSO	1.000	0.002	0.003	1.000	0.001	0.002	613.494	181.914	0.722
grSCAD	1.000	0.004	0.006	1.000	0.001	0.002	613.466	181.899	0.722
grMCP	1.000	0.005	0.007	1.000	0.002	0.004	613.229	181.898	0.722
ψ=0.4									
grLASSO	1.000	0.002	0.004	1.000	0.001	0.002	457.330	120.632	0.695
grSCAD	1.000	0.005	0.007	1.000	0.003	0.005	457.337	120.631	0.696
grMCP	1.000	0.004	0.006	1.000	0.002	0.004	457.337	120.630	0.696
ψ=0.5									
grLASSO	1.000	0.002	0.003	1.000	0.001	0.002	360.622	84.836	0.668
grSCAD	1.000	0.004	0.005	1.000	0.003	0.004	360.632	84.830	0.668
grMCP	1.000	0.004	0.007	1.000	0.002	0.004	360.419	84.803	0.668

## Appendix C Description of the German Healthcare Demand Data

**TABLE C1:** Description of predictor variables (stratified by groups) in the German healthcare demand data.

Group	Variable	Description
1	Age1, age2, age3	First, second, and third degree polynomial of age
2	Health1, health2, health3	First, second, and third degree polynomial of health satisfaction
3	Hdegree1, hdegree2, hdegree3	First, second, and third degree polynomial of the degree of handicap in percentage points
4	Schooling1, schooling2, schooling3	First, second, and third degree polynomial of years of schooling
5	Hhincome1, hhincome2, hhincome3	First, second, and third degree polynomial of household monthly net income
6	Handicap	1 if handicapped, 0 otherwise
7	Married	1 if married, 0 otherwise
8	Children	1 if children under 16 in the household, 0 otherwise
9	Self	1 if self‐employed, 0 otherwise
10	Civil	1 if civil servant, 0 otherwise
11	Bluec	1 if blue collar employee, 0 otherwise
12	Employed	1 if employed, 0 otherwise
13	Public	1 if public health insurance, 0 otherwise
14	Add‐on	1 if add‐on health insurance, 0 otherwise

## References

[1] S. Chatterjee , S. Chowdhury , H. Mallick , P. Banerjee , and B. Garai , “Group Regularization for Zero‐Inflated Negative Binomial Regression Models With an Application to Health Care Demand in Germany,” Statistics in Medicine 37, no. 20 (2018): 3012–3026. 29900575 10.1002/sim.7804

[2] H. Wang and C. Leng , “Unified LASSO Estimation by Least Squares Approximation,” Journal of the American Statistical Association 102, no. 479 (2007): 1039–1048.

[3] L. Dai , K. Chen , Z. Sun , Z. Liu , and G. Li , “Broken Adaptive Ridge Regression and Its Asymptotic Properties,” Journal of Multivariate Analysis 168 (2018): 334–351. 30911202 10.1016/j.jmva.2018.08.007 PMC6430210

[4] N. Li , X. Peng , E. Kawaguchi , M. A. Suchard , and G. Li , “A Scalable Surrogate L0 Sparse Regression Method for Generalized Linear Models With Applications to Large Scale Data,” Journal of Statistical Planning and Inference 213 (2021): 262–281.

[5] H. Zhao , Q. Wu , G. Li , and J. Sun , “Simultaneous Estimation and Variable Selection for Interval‐Censored Data With Broken Adaptive Ridge Regression,” Journal of the American Statistical Association 115 (2020): 204–216. 32742044 10.1080/01621459.2018.1537922 PMC7394486

[6] X. Su , J. Fan , R. A. Levine , X. Tan , and A. Tripathi , “Multiple‐Inflation Poisson Model With *L* _1_ Regularization,” Statistica Sinica 23 (2013): 1071–1090.

[7] J. Lv and Y. Fan , “A Unified Approach to Model Selection and Sparse Recovery Using Regularized Least Squares,” Annals of Statistics 37, no. 6 (2009): 3498–3528.

[8] L. Dicker , B. Huang , and X. Lin , “Variable Selection and Estimation With the Seamless‐*L* _0_ Penalty,” Statistica Sinica 23 (2013): 929–962.

[9] P. Breheny and J. Huang , “Group Descent Algorithms for Nonconvex Penalized Linear and Logistic Regression Models With Grouped Predictors,” Statistics and Computing 25 (2015): 173–187. 25750488 10.1007/s11222-013-9424-2 PMC4349417

[10] F. K. Hui , K. D. Dang , and L. Maestrini , “Simultaneous Coefficient Clustering and Sparsity for Multivariate Mixed Models,” Journal of Computational and Graphical Statistics 34, no. 2 (2025): 618–629.

[11] X. Zhu , F. Li , and H. Wang , “Least‐Square Approximation for a Distributed System,” Journal of Computational and Graphical Statistics 30, no. 4 (2021): 1004–1018.

[12] P. Zeng , Y. Wei , Y. Zhao , et al., “Variable Selection Approach for Zero‐Inflated Count Data via Adaptive Lasso,” Journal of Applied Statistics 41, no. 4 (2014): 879–894.

[13] E. O. Ogundimu , “On Lasso and Adaptive Lasso for Non‐Random Sample in Credit Scoring,” Statistical Modelling 24, no. 2 (2024): 115–138.

[14] F. Frommlet and G. Nuel , “An Adaptive Ridge Procedure for l 0 Regularization,” PLoS One 11, no. 2 (2016): e0148620. 26849123 10.1371/journal.pone.0148620 PMC4743917

[15] E. D. Schifano , R. L. Strawderman , and M. T. Wells , “Majorization‐Minimization Algorithms for Nonsmoothly Penalized Objective Functions,” Electronic Journal of Statistics 4 (2010): 1258–1299.

[16] R. J. Hyndman and A. B. Koehler , “Another Look at Measures of Forecast Accuracy,” International Journal of Forecasting 22, no. 4 (2006): 679–688.

[17] Z. Wang , S. Ma , C. Y. Wang , M. Zappitelli , P. Devarajan , and C. Parikh , “EM for Regularized Zero‐Inflated Regression Models With Applications to Postoperative Morbidity After Cardiac Surgery in Children,” Statistics in Medicine 33, no. 29 (2014): 5192–5208. 25256715 10.1002/sim.6314 PMC4227915

[18] G. Shmueli , “To Explain or to Predict?,” Statistical Science 25 (2010): 289–310.

[19] S. Ciavarro , Group Regularisation for Zero‐Inflated Poisson Models. Unpublished BSc dissertation (Department of Mathematical Sciences, Durham University, 2024).

